# Facilitating perspective-taking with animals in human decision-making

**DOI:** 10.1017/awf.2026.10078

**Published:** 2026-03-26

**Authors:** Erin B. Ryan, Matthew I. Billet, Daniel M. Weary

**Affiliations:** 1 The University of British Columbia, Canada

**Keywords:** Animal welfare, cow-calf separation, decision-making, empathy, farm animals, human-animal relationships, perspective-taking

## Abstract

Efforts to include animal perspectives in decision-making are gaining attention, yet how to meaningfully represent these perspectives remains underexplored. This study investigated how university students engaged in taking the perspective of dairy cows and calves when introduced to the practice of cow-calf separation — either through a verbal description or a visually immersive video capturing the animals’ point of view. Focus groups were conducted to examine the range and depth of participants’ responses, and transcripts were thematically analysed. Results revealed that participants across both treatments acknowledged the animals’ experiences, particularly the emotional significance of the maternal bond. However, those exposed to the video condition engaged in more emotionally detailed and complex discussions, often referencing specific animal behaviours and vocalisations. The video appeared to enhance perspective-taking by increasing contextual richness, encouraging participants to interpret the animals’ experiences more vividly. While many participants expressed empathy or sympathy, others reported distress or hesitancy, citing challenges, such as anthropomorphism or uncertainty about accurately accessing animal perspectives. These findings underscore the potential for visual interventions to deepen understanding of non-human perspectives, while also highlighting psychological and cultural barriers to animal-inclusive decision-making. Our results suggest that perspective-taking can be a valuable tool in promoting ethical engagement with animal welfare. However, further research is needed to explore how such engagement influences actual decision-making, and how to balance emotional connection with critical reflection.

## Introduction

Decisions regarding the use and treatment of animals are often made without considering their perspectives, but there is a growing call for their inclusion (Donaldson & Kymlicka 2023; Thomsen *et al.* 2023). Efforts to expand governance frameworks to include non-human interests offer protections for the environment and reflect a Rights of Nature approach. These models have primarily addressed the rights of ecosystems or broader natural entities, such as the Te Awa Tupua River in New Zealand to whom legal personhood status was granted (for an overview of the legal personhood granted to the Te Awa Tupua river, see the National Library of New Zealand (n.d.) but there is also a need to consider the perspectives of individual animals. Rights of Nature frameworks often assume that the interests of ecosystems align with those of all the individual animals contained therein, but this assumption is not always true and does not clearly apply to the context of domesticated species such as farmed animals, which represent a large proportion of the mammalian biomass (Ritchie & Roser 2021; Schneider 2022). While efforts to represent the interests of animals in policy are gaining traction, *how* to include these individual perspectives is not well understood. Innovative approaches, such as the use of AI to better understand animal vocalisations (Hagiwara *et al.* 2023; Miron *et al.* 2025) and virtual reality experiences to better understand the animal’s perspective (Ahn *et al.* 2013, 2016; Bailenson 2018; Herrera *et al.* 2018) show promise, but these approaches have not yet been applied in decision-making contexts. For effective animal-centric decision-making, decision-makers must find ways of assessing the animal’s perspective and including this in their outcomes. Such efforts are challenged by psychological barriers, anthropocentric biases, and a disconnection with nature that characterises contemporary urban living in many societies (Ahn *et al.* 2016; Cornips & van den Hengel 2021; Donaldson & Kymlicka 2023).

### What is perspective-taking and why does it matter?

Perspective-taking is the cognitive process of mentally adopting or understanding another individual’s point of view by imagining their thoughts and feelings (Davis & Love 2017). This cognitive process can be accompanied by an emotional component, in which one empathetically shares in the emotional states of others (Davis & Love 2017), as well as a motivational component, in which one feels sympathetically motivated to alleviate the distress of others (Wispé 1986; Singer & Klimecki 2014). These responses can increase feelings of closeness to others (Sevillano *et al.* 2007; Ahn *et al.* 2016) and even promote a sense that one shares an overlapping identity with others (i.e. self-other overlap; Schultz 2000, 2001). Fostering closeness to others through perspective-taking has been associated with altruism towards others (Eisenberg 2006), even bridging the distance between people of different social and racial groups (Fussell & Krauss 1989; Batson *et al.* 1997).

While research has generally linked perspective-taking to positive outcomes, this is not always the case. When another’s distress is upsetting, the motivation to alleviate your own empathetic distress may be greater than that to alleviate the other’s distress (Batson *et al.* 1987). In such cases, perspective-taking may backfire by prompting avoidance or disengagement from the other’s suffering; this effect may be especially likely in cases where the other’s experience is particularly negative (as may occur with non-human animals; Batson *et al.* 1987).

Psychological research on perspective-taking has largely focused on the behavioural effects on humans taking the perspective of other humans (e.g. Todd & Galinsky 2014), but the same mechanisms that connect perspective-taking to altruistic outcomes in human-to-human contexts — empathic concern and self-other overlap — may also be implicated when taking the perspective of non-human animals (Berenguer 2007; Schultz 2007; Sevillano *et al.* 2007; Ahn *et al.* 2016; Auger & Amiot 2019; Ladak *et al.* 2023). Ladak *et al.* (2023) asked participants to take the perspective of farmed pigs and found that these participants reported greater empathic concern and self-other overlap with the farmed pigs compared to control conditions. Berenguer (2007) asked participants to view images of birds or trees that had been harmed and found that participants in the perspective-taking condition reported greater empathic concern and a greater willingness to allocate resources to environmental protection compared to a control condition. Other studies have relied on more immersive procedures to enhance the impact of an intervention, like one study that found that immersive virtual environments simulating sensory-rich animal experiences increased feelings of embodiment and interconnection with nature, while also heightening perceptions of environmental risk (Ahn *et al.* 2016).

Understanding animal preferences and motivations form a foundation for promoting perspective-taking between humans and animals, leading to questions regarding what animals value and allowing for better insights into the animal’s experience. Animal welfare scientists have developed a range of methods to better understand the preferences of animals (for a review, see Franks 2019). These methods include assessing animal preferences and motivations using behavioural and physiological measures (Balcombe 2009; Weary *et al.* 2017; Fife-Cook & Franks 2019; Špinka 2019). Conceptual work has begun to consider how insights derived from these studies can be used to improve the conditions in which animals live (Špinka 2019; Weary & Robbins 2019).

A number of barriers can prevent people from considering the perspectives of non-human animals. Many people rarely see or share experiences with non-human animals, which makes their experiences and perspectives less salient and less accessible. Broader anthropocentric cultural norms that prioritise human perspectives and de-emphasise animal agency may also contribute to a tendency to disregard non-human animal perspectives (Meijer 2019; Cornips & van den Hengel 2021; Donaldson & Kymlicka 2023). Finally, it is uncomfortable to empathise with other’s suffering, and people may be motivated to avoid engaging with the perspectives of non-human animals that are subjected to poor treatment (Batson *et al.* 1987). Although images of environmental harm can promote empathic concern (e.g. Berenguer 2007), potential perspective-taking interventions that expose participants to animal suffering may backfire by promoting avoidance. However, little is known about how participants process these experiences. Most research to date relies on quantitative outcomes, such as changes in reported empathy or self-other overlap, without examining how people articulate or make sense of their experience. Understanding these subjective experiences can help identify not only backfire effects like avoidance, but also novel approaches that could enhance the design of animal-centred interventions. In particular, comparing less immersive interventions (e.g. text-based descriptions) with more immersive ones (e.g. audio-visual footage) may reveal differences in how deeply participants take the animal’s perspective, and what barriers they encounter. Qualitative methods such as focus groups are well-suited to explore these dimensions.

The aim of the current study was to explore how university students engaged in taking the perspective of dairy cows and calves when encountering the practice of cow-calf separation shortly after birth (Meagher *et al.* 2019). We examined how animal perspectives were incorporated into participants’ discussions during focus groups, following their exposure to either a verbal description or a video of the practice, with the video intentionally framed to capture the animals’ point of view. A secondary aim was to examine variation in participants’ engagement in animal perspective-taking, with the two exposure methods (description vs video) used to help increase this variation.

## Materials and methods

Psychology undergraduate students, over the age of 18 years, were recruited for this study through the University of British Columbia (UBC) Psychology Department’s Human Subject Pool (HSP). Participants were informed that data collected would not personally identify them, that they were free to leave the study at any point and were provided information about data storage. Participants provided their consent to participate before beginning the study. They were also given a link to a survey to complete; the results of this survey are not described here but the full survey text, question guide, anonymised data, and the NVivo files are publicly available (https://borealisdata.ca/dataset.xhtml?persistentId=doi:10.5683/SP3/J6JG9D). In exchange for their time, participants received 0.5 course credits for every half hour of their participation. For this study, which was estimated to take no more than 1 h 15 min, participants were compensated with 1.25 credits. The course credits were allocated to each participant via UBC Psychology Department’s Human Subject Pool portal. This study was approved by the University of British Columbia’s Behavioural Ethics Review Board (ID #H23-01272).

### Study design

Participants were interviewed in small groups and asked to take the perspective of the animals involved in cow-calf separation. A total of 51 participants were recruited into 12 focus groups, each consisting of 2–6 people. A fixed target sample size was not set *a priori.* Consistent with qualitative research norms, sample size was guided by feasibility and thematic saturation rather than statistical power. Focus groups were exposed to two different treatments: one that received an auditory description of cow-calf separation, based on the National Farm Animal Care Council Dairy Code (2009), while the other watched a 22-min video segment from the documentary “Cow” (Arnold 2021), which provided a visual and auditory representation of the separation from the cow and calf’s perspective. Focus group sessions occurred sequentially as participants signed up for the study. Treatment order was randomly assigned within each pair of consecutive sessions using a coin toss.

Focus groups were conducted on Zoom (Zoom Video Communications Inc, San Jose, CA, USA 2021), between July and October 2023. Each session lasted approximately 2 h, beginning with an online survey that took approximately 10 min to complete.

### Focus groups

In all focus groups, participants were introduced to the practice of cow-calf separation using a text read aloud by ER:

*“Cow-calf separation is an issue that is receiving attention from the public, animal welfare scientists and farmers. Standards are set for the 10,000 dairy farms Canada (with a total of about 1.4 million dairy cows) by the National Farm Animal Care Council (NFACC) Code of Practice for the Care and Handling of Dairy Cattle (Dairy Code). The practice of cow-calf separation is addressed in the 2009 code but not in the most recent 2023 code. It receives updates, based in part, on public comments. Today, I want you to imagine that you’ll be commenting on this practice.”*

Half the groups had a description of the practice of cow-calf separation, taken from the NFACC Dairy Code (2009) read aloud to them, by ER:

*“Generally, dairy calves are separated from their mothers shortly after birth. There are benefits to both calf and dam by allowing the pair to bond. Allowing the calf to spend a longer period of time with the dam may result in lowered morbidity and mortality in the calf; however, separation stress to both the cow and calf will be higher the longer they are together. Cow health is generally improved by allowing the calf to suckle* [related to oxytocin effects on the post-partum uterus]*. Whether the calf is removed immediately or allowed to suckle the cow, it is important to ensure that the calf receives adequate colostrum.”*

Video treatment groups were provided licensed access to Andrea Arnold’s documentary film “Cow” (Arnold 2021). Participants were asked to watch a 22-min clip (time-stamp from 0:28–22:26 min mark) of the film. The film uses low and close-up camera angles to capture the cow’s and calf’s perspective of being separated from one another following the calf’s birth. While farm workers were occasionally in view, their faces were not featured, no dialogue was included, and there was no voice-over narration. The only sounds that viewers heard came from the animals themselves and ambient sounds from the barn (e.g. music in the milking parlour, sounds of machinery). The film segment that participants watched began with the birth of the calf, through to the separation of the calf from the cow, and following the separation, the film focused on the cow’s (Luma) expressions and some footage of her calf in an individual hutch. Before watching the video, the facilitator said to participants “*try to consider the experience from the point-of-view of Luma, the cow, and of her calf. Really watch all the communication coming from Luma and her calf, listen closely to vocalisations and other expressions while you try to consider the experience from their perspectives*”.

After the intervention, groups were asked whether or not they had ever heard of the practice of cow-calf separation, their initial thoughts on the practice, whether or not they could take the perspectives of the animals involved, what questions they had that would help inform their views on the practice, what from the focus group conversation stuck with them, what surprised them, and what they learned from the conversation. No definition of perspective-taking was given to participants. ER acted as group facilitator, guiding the discussion. DW also participated during the first five groups, answering questions asked by participants as required on technical aspects and the related science; after these initial sessions it became clear that ER was able to answer these questions.

### Analysis

Otter.ai was used to transcribe focus group conversations into transcripts and NVivo (version 14.23.1; QSR International, Burlington, MA, USA) was used to analyse transcripts. Qualitative analysis of the transcripts was conducted at the group-level and focused on understanding the ways that participants described taking the perspectives of the animals, including language they used that reflected concern for the animals’ experience. Analysis was not focused on accuracy of participants’ perspectives. Rather, coders sought to understand themes related to the participant’s efforts to perspective take.

To develop a codebook and ensure inter-rater reliability, three transcripts were randomly selected (two from the video treatment and one from the audio treatment). Two coders (ER and research assistant, C Kuo) independently coded the first transcript, focusing on how participants expressed perspective-taking regarding the animals involved in cow-calf separation. During this process, the coders applied descriptive codes to participant dialogue, using nouns or tags to capture key elements (Saldaña 2020). These codes were then grouped into broader thematic categories. After coding the first transcript, the coders met to compare their coding and discuss any discrepancies.

The process was repeated for the second transcript, with the coders adjusting codes as necessary. By the time they coded the third transcript, the coders had reached a high level of agreement, indicating theme saturation (Morse 2015). ER used the final codebook to complete coding of the remaining transcripts. Quotes presented in the results were selected to best represent the theme. Citation of quotes included the pseudonym chosen by the participant (shown within the text), e.g. “Sarah” or “C” (some participants chose only one letter pseudonyms) and in square brackets the focus group number assigned to their group (e.g. FG2), and the treatment this group was assigned to audio or video, for example [FG9, audio].

## Results

### Sample description

Participants ranged in ages from 18 to 29. Forty participants were female, 10 were male, and 1 identified as non-binary. Forty-nine participants described themselves as omnivores and two as vegetarian. The number of participants per focus group (i.e. 2–6 participants) was similar for the two treatments, showing a mean (± SD) of 4.0 (± 0.6) vs 4.5 (± 1.6) participants for audio and video treatments, respectively.

### Thematic results

Our results show that most themes were consistently observed across groups, consistent with saturation. Participants’ efforts to take the perspectives of animals involved in cow-calf separation are described through four themes: Acknowledging the animals’ experience; Seeing/hearing the animals; Relating to the animals’ experience; and Challenges to taking the animals’ perspective (Table 1). Sub-themes are presented with varying lengths of quotes to capture the range of language used and to represent the data effectively. Participant comments could encompass multiple themes, and participants who watched the video often referenced seeing and hearing the animals, resulting in shorter quotes in sub-themes unrelated to this treatment. Additionally, sub-themes in which participants from one treatment provided more comments are illustrated with more quotes.

**Table 1.** Themes derived from transcript analysis of focus group (n = 12) assigned to two treatments: an audio description of cow-calf separation, or a film clip depicting the experience of a cow and calf. Comments could be valanced positively or negatively (e.g. being able to take the perspective of animals or not) and could include multiple themes

Theme	Summary of theme	Subthemes
Acknowledging the animals’ experience	Participant recognises that animals are having an experience	-Acknowledging the experience of the maternal bond - Speculations on the experience of animals
Seeing and hearing the animal(s) (Video groups only)	Interpreting the physical and vocal expressions of individual animals	-Emphasising animals’ perspectives -Recognising individual experiences
Relating to the animals’ experience	Expresses empathy regarding the animal’s experience	-Emotional impact on participants -Getting in the animals’ ‘shoes’ -Seeing similarity to animals
Challenges to taking the animals’ perspective	Hesitancy, scepticism towards, or uncertainty about taking the animals’ perspective. Recognition of elements that limit the ability for animals to express their interests and act upon these	-Lack of experience, knowledge, or understanding -Barriers to understanding the animal’s perspectives -Limited view on the capacity for animals to have a meaningful perspective -Restricted environments reduce animals’ ability to express behaviours -Understanding what matters to animals

### Acknowledging the animals’ experience

This theme included comments relating to being able to recognise the perspectives of animals. Participants specifically identified with the maternal bond between cows and calves, emphasising its unique emotional significance in the context of cow-calf separation, and where speculation was made in reference to the animals’ emotional, physical, or cognitive experience of having that bond broken. This theme was articulated often and by all groups (Table 2).

**Table 2.** Prevalence of five themes in the discussions of 12 focus groups exposed to either an audio description or a video depiction of cow-calf separation. Indicated is the number of focus groups (Grp; max of 6 in each treatment) that addressed this theme, and the total number comments (Tot) that referenced the theme, separately for the two treatments

Treatment	Acknowledging the Animals’ Experience		Seeing/Hearing the Animal		Relating to the Animals’ Experience		Challenges to Taking Animals’ Perspective	
	Grp.	Tot.	Grp.	Tot.	Grp.	Tot.	Grp.	Tot.
Audio	6	43	0	0	6	19	4	8
Video	6	53	6	48	6	52	6	40

#### Acknowledging the experience of the maternal bond

We asked all participants if they were able to take the perspectives of these animals. In response, participants often acknowledged the experience of the maternal bond between cow and calf and what it would be like for animals to have this severed, including how they would experience the separation. For example, ‘C’ [FG10 video] said, *“I don’t know a lot about cows, but I know they are mammals. So they should have some connection to their children…I guess I can sort of identify with that as a mammal.* Another participant [Jane; FG1 video] described cows and calves as *“biologically programmed”* to be in a caring relationship and that fear and confusion would result from separation. Some people saw the cow-calf bond as similar to that experienced by other animals; ‘Raine’ [FG13 audio] stated *“…[T]his reminds me of the relationship between a human mother and her child because I remember something like…the earlier the child is separated from their mother* [the more likely] *they will be anxious and feel like a lack of security in the future and I think maybe the same situation will be applied to the calf and its mother if they are separated very early, maybe at birth…maybe they will feel unsafe or anxious*”. Other participants like ‘Rebecca’ [FG11 audio] stated that she felt that the experience may *“not… be the same as what a human would experience”*, but that she could *“imagine that there’s a lot of feeling surrounding the entire experience for the calf because* [they] *are new to the whole world and the one point of familiarity* [they] *do have is being taken away. And I’m also imagining that there’s some sort of distress on the mother’s side”.* These responses underscored a recognition of the maternal bond between cows and calves, with many identifying the emotional toll of separation and drawing comparisons to human parent-filial relationships. Some participants highlighted the unique perspective of the calf in its unfamiliar environment, suggesting a nuanced understanding of the experience.

#### Speculations on the experience of animals

Participants in both treatments were able to describe the perspectives of cows and calves in general terms, and some participants (especially those in video treatment groups) speculated on a range of factors including the animals’ experience of artificial maternal substitutes, dependency on humans, and their experience of trust in humans. For example, ‘Woof’ [FG 4 video] shared that a calf “*trying to nestle itself*” up to a plastic milk bottle in their hutch, wouldn’t be able to *“find comfort in it.* Participant ‘Richard’ [FG8 video] considered what the experience of separation would be like for the cow, and expressed his feeling that separation relies on *“a lot of psychological tricks”* being *“play*[ed]*”* on the cow and calf, saying, *“the calf doesn’t even get milk from* [their] *mother, it gets milk from* [a] *human, and it’s a way of isolating the calf from the mom, and kind of automatizing them…I don’t think animals feel intentions and emotions very differently from humans …I think that the mother would probably just feel increasingly depressed every moment along the way”.*

In another group, ‘Fred’ [FG8 video] shared his insights into animals’ experiences of trust and potential impact across generations. Specifically, he questioned the breaking of bonds between animals and how this might affect trust in human handlers:

*“… this calf is now going* [to go] *through this entire process, where it’s being raised to eventually also give birth, and then having its child taken away…it’s like the handlers are sort of taking the place of a mother where they’re feeding it*…[and are]…*the people you’re trusting to provide for you, even if it’s not obviously the greatest way to live. That’s the only life they know”.*

Fred went on to highlight the potential complexity of downstream effects, in this case asking what it would be like to rely on the same person who removed you from your mother, and who will remove your calf from you:

*…And now those same people are taking away your child, who you obviously are going to have very strong emotions for… So it’s kind of like confusion, again, like they don’t really know why it’s happening, but they’ve trusted these people…and now they’ve taken* [that trust] *away. And then I feel like that might tie into…well, we don’t know how much awareness they have but, would they be aware that now this is going to happen to their baby essentially? … I don’t know if that shifts how they respond to the handlers, but it almost seems like it could be a point of broken trust as well as confusion.”*

Thus, in these instances, speculating on the experiences of animals extended beyond general acknowledgment to include specific considerations of artificial maternal substitutes, forced dependency on humans, and the erosion of trust, suggesting a more nuanced attempt at understanding what separation means to the cows and calves.

### Seeing and hearing the animal(s)

Comments included in this theme referred to the effect of the film on the participant’s ability to understand animals’ experience, and descriptions of participant’s views of the individual experiences of the animals, including interpretations of Luma and her calf’s body language, behaviour, and vocalisations. Not surprisingly, participants exposed to the video often made comments that fit within this theme; in contrast, none of the comments from participants in the audio treatments were captured in this code.

#### Emphasising animals’ perspectives

The film’s emphasis on the animals’ perspectives was commented on by many participants, who talked about how it affected their perspective-taking. For example, participant ‘E’ [FG10 video] said: *“how it was filmed and* [seeing] *the perspective of the cow… really made me think about how it feels* [for] *the cows”.* Another participant, ‘Cat’ [FG4 video] said: *“I sort of liked how there was no* [human] *dialogue because cows can’t talk…* [the film makers] *could have had someone explaining what was going on but you didn’t need that because you could just so clearly see the emotions from both of the cows: the mother and the daughter. So, I think it just emphasized that they do have emotions and feel the same things we feel.”* Similarly, ‘Anna’ [FG12 video] articulated the difference that she felt between being told about a practice versus seeing it for herself, saying:

*“*[The video is] *not just telling you about it, you’re not just reading about it, you’re actually seeing it with your own eyes. And I think through this way, it makes you feel for the animals a little bit more. And it makes you feel like you can connect with them emotionally. And I think that’s what we were all feeling a little bit, we could see like the stress in the mother’s eyes, we could feel that the stress when the calf was being* [ear] *tagged as well.”*

Here, participants’ reflections on the videos portrayal of animals’ perspectives highlighted the impact of visual storytelling in fostering empathy and understanding, emphasising the emotional connection established by witnessing the animals’ experiences first-hand rather than relying solely on a verbal description.

#### Recognising individual experiences

Other participants described how the film allowed individual animals to express themselves, including through their vocalisations and body language. For example, ‘Jane’ [FG1 video] said: *“I feel like they’re calling out for their mom or their calf, and they’re trying to let them know that they’re there or trying to find them almost.”* Participant, ‘KB’ [FG5 video] felt that Luma *“was crying”* for her calf and trying to express that she wanted to get to *“her child”*; that Luma and her calf were *“depressed because they were not able to be together and neither of them were getting a family.”* Other comments illustrated how what participants saw led them to consider nuanced expressions of the animals’ perspectives. For example, ‘Susan’ [FG8 video] observed that she *“saw that near the end of the video, there was a scene where they were all eating but* [Luma] *refused. She wasn’t eating, she was just standing there. And I thought, that’s kind of like, that’s one of those signs that shows you that she’s being affected by this. The mother is being affected by this – her calf being taken away…it’s obvious that she’s showing emotions, she’s showing grief in her own way.”* Here, the participants’ comments highlight the video’s portrayal of individual animals expressing themselves, particularly through vocalisations and body language. Witnessing these expressions prompted reflections on the animals’ emotional experiences. For instance, participants observed how Luma expressed her grief.

### Relating to the animals’ experience

The theme of ‘Relating to the animal’s experience’ was derived from comments where participants referenced ways that they empathised with the animals, either by sharing their own emotional reactions to the practice of cow-calf separation as a way of relating to animals’ experience of being separated from one another, by articulating their attempt to imagine themselves in the animal’s position either by speaking for the animal or as though the person was the animal, or where they related to the animal’s experience. This theme was present in all groups but appeared more frequently in comments by participants in the video treatment.

#### Emotional impact on participants

Participants in all focus groups described their own emotional response to the animals’ experience. Participants in audio groups often used simple language; for example, ‘Anne’ [FG 9 audio] said *“it’s really sad how all these farmers are trying to separate them”*, and ‘Omi’ [FG 13 audio] said that cow-calf separation was *“obviously very sad to hear* [about]*”.* In contrast, participants exposed to the video treatment provided more detailed descriptions of their feelings. For example, video treatment participants described feelings of *“shock”* at encountering the practice [‘Amara’ FG10 video] [‘Susan’ FG8] [‘Ivy’ FG12 video] [‘Quack’ FG4 video] or feeling *“weird”* [‘S’ FG10 video], *“disturbed”* [‘Rose’ FG8 video] and *“uncomfortable"*, and that the practice was *“jarring”* [‘Susan’ FG8 video] or *“brutal”* [‘Sharn’ FG4 video] to see. Others described feeling *“upset”* [‘NM’ FG5] and experiencing *“psychological discomfort”* [‘Eileen’ FG12 video] after watching the video. Thus, seeing the animals seemed to provoke a stronger and more negative response for participants, although all participants expressed some degree of sadness about the practice.

#### Getting in the animals’ ‘shoes’

Participants showed empathy for cows and calves by attempting to imagine what the animals were experiencing, using their imagination to see the perspective of the animal. Participant ‘E’ [FG10 video] said: *“If I would put myself in the cows’* [position] *right now, I would feel horrible, terrible…”* or as ‘Jane’ [FG1 video] said, *“If I were to put myself in their shoes, but from a human perspective, I think that* [separation] *would be horrible to go through.”* While expressions of empathy were generally more limited in the audio treatment, participant ‘Jack’ [FG9 audio] described how he empathised with animals experiencing cow-calf separation:

*“[W]henever people talk about situations, the first thing you kind of do instinctually is just* [try] *to imagine yourself in that situation. I don’t think it’s any different for animals. Like, I just think, if you just imagine you’re just born, and then immediately you’re like, taken away and you don’t really know why or understand, you don’t know anything. That just seems like a very heartbreaking thing to have to go through regardless of* [whether] *it’s an animal* [or] *it’s a human.*”

Thus, through their efforts to connect with the perspectives of cows and calves, participants sought to understand the animals’ experiences by envisioning themselves in the animals’ positions, revealing a connection between human imagination and empathy towards animal perspectives.

#### Seeing similarity to animals

Seeing the similarity between the experience of a cow and calf being separated to that of a human mother and child being separated was one way that participants attempted to understand the experience of cow-calf separation. Participant, ‘Rose’ [FG8 video] said: *“I feel like something that would stick out to me is just the lack of happiness that these animals feel like it feels like such a contrast to our lives. Like it seems like the calf and the cow will never know a happy day in their lives.”* In another group, ‘Alicia’ [FG3 audio] concluded that the calf’s experience of being separated from its mother would result in *“a negative emotion”* with *“life changing effect*[s]*”*, arriving at this appraisal by *“imagining* [herself] *having to separate from* [her] *parents.”*

In these comments, a different emphasis emerges compared to the earlier sub-theme of *Acknowledging the experience of the maternal bond*, where participants emphasised the emotional significance of the bond between cows and calves. Here, participants draw parallels between the experiences of cows and calves and those of humans, highlighting similarities between the two as a means of connecting to the animals’ experience.

### Challenges to taking the animal’s perspective

Participants in both treatments commented on barriers in being able to take the perspectives of animals, including uncertainty about their ability to access these perspectives, uncertainty about the accuracy of interpretations of animals’ expressions of subjectivity, scepticism around the ability of animals to have meaningful perspectives, challenges people perceived related to restrictive environments reducing animal’s ability to express behaviours, and challenges associated with understanding what animals want. This theme arose in 4 of the 6 focus group sessions for both treatments. More comments were coded for this theme in video groups, a result driven by one group [FG10] that commented more on these challenges.

#### Lack of experience, knowledge, or understanding

When asked if they could imagine the experience of cow-calf separation from the cow’s perspective, some participants expressed difficulty, finding it challenging to imagine, while *others* felt they lacked experience or knowledge, or that species differences were too significant to overcome. Participant ‘Alice’ [FG11 audio] shared her frustration when asked to take the perspectives of animals involved in cow-calf separation, saying, *“Ah…aye aye* [strained expression]*, I have been trying – um…I’m not sure how to imagine the experience…like, just to imagine the cow as human, maybe like this way?”.* ‘Haha’ [FG11 audio], felt that the task of evaluating *“how animals perceive things”* might be better suited to people with animal expertise. For ‘Emily’ [FG13 audio], the struggle to take the perspective of animals rested in part with her lack of experience in *“really think*[ing] *much about the perspective of animals”.* She elaborated saying, *“when I was asked that question, it kind of caught me off guard. I think a lot of time we are so occupied that we don’t really think of the animal’s perspective.”* For ‘Richard’ [FG8 video], seeing the film made him reflect on how not encountering the experiences of farm animals creates challenges to being able to respond to their perspectives. He said, “*Humans are really good at putting stuff in the back of our minds…we just sort of block it out…just so we can…put some cream in our coffee…*[we] *block out just a bunch of horrific stuff we do to living things…just so we can have minor conveniences, minor pleasures in our own lives.”*

In summary, participants’ success in imagining the cow’s perspective on separation varied, with some facing challenges due to a lack of experience or difficulty reconciling species differences, and others feeling that there was a need for specialised knowledge relating to animal perception. Richard’s comment highlights how exposure to information about farm animals’ experiences can reveal cognitive dissonance regarding human treatment of animals, emphasising the tendency to overlook discomforting realities.

#### Barriers to understanding animals’ perspectives

Even when participants attempted to take the perspectives of animals some, like ‘Jane’ [FG11 audio], voiced hesitancy regarding the ability of people to understand these perspectives, saying *“we can never completely understand what animals would think because we are different species.”* Participant ‘Sania’ [FG3 audio], questioned if any methods exist that could be used to *“actually talk to cows, to know their perspective?* In one focus group, ‘Steve’ [FG15 audio] argued that it was *“dehumanizing”* to use the lack of language as a reason for not attempting to understand the cows’ preferences. He remarked *“[I]t seems like the cows do not have a say in their own lives just because they don’t communicate the way we communicate*”. Steve’s point is that the lack of verbal, human, communication shouldn’t be a barrier to acknowledging and seeking to understand animals’ experiences and preferences.

Concerns regarding an anthropocentric bias (e.g. concern on the part of participants that any perspectives they were perceiving in the animals were simply a result of their own human projection on to the animal, and perhaps not what the animal was actually feeling) in interpreting animal experiences were voiced in other groups. Participant ‘Selena’ [FG9 video] shared her concern around misinterpreting the animal, saying: *“*[When] *we are watching the video* [are we] *actually just projecting ourselves onto that animal.”* Participant ‘C’ [FG10 video] felt that cows having *“different cognitive abilities, and different abilities in general”* made it difficult *“to actually get a proper representation of what a cow would be…because we will always do it from our own humanness and centrism and [from] our own perspective…”.*

Participants’ comments here point to the concern they felt toward interpreting animal experiences without projecting human perspectives onto them.

#### Limited view on the capacity for animals to have a meaningful perspective

Some participants saw the behaviour of Luma and her calf as ‘just biology’ or responses either not reflective of a perspective or revealing a very limited perspective. This view was pronounced in one group [FG10 video]. For example, ‘Amara’ [FG10 video] expressed, *“I feel like … that it’s more of the biology, the motherly instinct of mammals and animals that they have connected to when they give birth, but then I feel a little after that moment of separation, they’re just going to keep…continuing on without really feeling anything until they have to give birth to* [another calf]*.”* ‘L’ in the same group, voiced doubt in our ability to understand anything about outward expressions from animals, sharing her belief that people could not understand the perspectives of animals and that perhaps that animals were experiencing little if any emotional response:

*“I was reminded of the Pavlovian conditioning experiment with the dogs and the salivating bells in the sense that…if the cows have never been exposed to anything that really, for example, helps wire certain neurocircuitry that allows for* [a] *dopamine response ahead of time, then their expectations are set very low, or low in the way that we as humans understand freewill. So, they’re not really experiencing anything other than what is meeting their expectations. So, even though we might be interpreting it as distress, they might be very content. I don’t really think that there’s a way with our current knowledge of neuroscience of understanding the experience they’re going through, I think everything is speculation based off of our frame of mind.”*

Here, participants expressed scepticism regarding the animals’ experiences, viewing behaviours as automatic rather than reflective of felt emotions. This perspective, exemplified by ‘Amara’ and ‘L’, is consistent with a mechanistic view of animals. ‘L’ suggested that her perspective was consistent with scientific knowledge on animal emotions, a point we shall return to in the *Discussion.*

#### Restricted environments reduce animals’ ability to express behaviours

Participants referenced factors they believed limit the ability of animals to express their interests or for those expressions to be recognised. These included restricted environments reducing animals’ ability to express behaviours (e.g. through physical restraint, limited movement, or through a practice, such as restricting the cow’s ability to express choice). This sub-theme emerged in two of the focus groups exposed to the audio treatment, and in all six of the groups exposed to the video. More participants in the video groups were able to comment on the perception of animals’ ability to express their interests likely because of visual affordances; they were able to see where animals’ ability to express themselves and make their own choices were frustrated.

In both groups, participants expressed concern for how the farm conditions may interfere with the animals’ ability to express their internal states, rendering animals vulnerable. Among audio participants, ‘Steve’ [FG15 audio] said he thought separating cows and calves was *“dehumanizing”* and ‘Sisilia’ [FG9 audio] spoke of animals being treated as instruments, such that farms were *“just using* [the animals] *as a tool to get profit”.* ‘Selena’s’ [FG1 video] said: *“*[the cow’s] *whole life* [is] *actually totally controlled by humans…Humans constrain them in very small places, and they offer food to the cows, but they did not ask what the cows really need, or what the cows really want, and they treat the cows just like an object – like an item, and they presume their interests. That’s why, if I were Luma* [the cow in the video clip] *I would say: I have no freedom.”* In the video groups, this theme was often expressed as a concern regarding a lack of agency (i.e. ability to exercise control over their own lives; Špinka 2019) and participants connected this thwarting of control to their perception of the animal’s perspective. For example, ‘Sharn’ [FG4 video] said, *“I think it must be pretty frustrating for* [Luma] *to give birth and then in the same day be milked by machines but not be able to feed her own child…the one thing that she wants to do”.* ‘Bob’ [FG8 video] brought up his perception of the cows’ feelings of *“having no control”* over the removal of their calf, while ‘Gibby’ [FG8 video] shared her opinion that isolating the calf and forcing dependency on humans was a way of *“automatizing”* animals.

In these comments, participants described how restricted environments can limit the animals’ ability to express their behaviours and interests, often perceiving animals as objectified or instrumentalised within the farming system. They highlight issues such as physical restraint, limited movement, and the restriction of choice, all of which compromise animals’ ability to express themselves. In the video groups, participants often had more vivid descriptions of the animals’ environments, likely because they were able to observe these directly.

#### Understanding what matters to animals

Many participants sought information about the experience of cows and calves, suggesting that people wanted to know more about the animals’ perspectives, and that this knowledge might affect their attitudes. For example, ‘Sisilia’ [FG9 audio] wanted to engage in the perspective-taking exercise, but felt that she needed more information about cows’ interests:

“*I’m just wondering if they’re aware of their family? Does it really matter if they need to stay with their family, like humans* [do]*? I think that’s important* [to know] *because I’m trying to think from the cow’s perspective…so, I’m not sure…do they have separate emotions…like humans? Does separation matter to them? If it does then…I think it’s heartbreaking.*”

Sharing her belief that a lack of understanding of animals’ perspectives could help account for why the practice of cow-calf separation continues, ‘Alice’ [FG11 audio] said, *“I think* [the practice] *is not fair but it’s very common on many farms because people don’t understand the animals’ perspective*”.

These comments reflect a desire from participants for insights into the experiences of cows and calves, and a recognition that a lack of understanding is contributing the ongoing practice of cow-calf separation. Additionally, they suggest the value of greater transparency in animal agriculture, as greater visibility could help bridge any gap between what is understood about animals’ capabilities and how they are cared for.

## Discussion

### Key results

This study explored how the perspectives of cows and calves were described by participants who encountered the practice of cow-calf separation, either by having a description read aloud to them or viewing a video showing a calf being separated from a cow. These treatments were not intended to test for differences between conditions, but rather to introduce variation in how participants experienced the practice, allowing us to examine a broader range of responses to animal perspective-taking. All focus groups acknowledged the animals’ perspectives, most notably through recognition of the importance of the maternal bond and the stress and confusion separation created in animals. Descriptions of animals’ perspectives were more prevalent, and were discussed more extensively, by participants who viewed the video; seeing and hearing the animals seemed to influence the depth and quality of discussions. In particular, these participants described in greater detail their emotional experience of encountering the cow and calf’s perspectives and it seemed to promote insights into how contextual elements impacted the animals’ ability to express their perspectives. Challenges in taking the animals’ perspectives were discussed in both treatments, but more of the participants in the video treatment raised these challenges. Overall, discussions in all themes received more engagement (as indicated by the number of comments), from participants who watched the video, suggesting that watching facilitated discussion and in some cases perspective-taking.

### Empathetic, sympathetic, and distress-avoidance reactions to animal perspective-taking

Participants exhibited a range of reactions to the perspective-taking exercise that have been identified earlier studies. Many widely expressed empathetic acknowledgement when imagining cow-calf separation, a response that aligns with Batson and colleagues’ work (1997), which shows that “imagine-self” perspective-taking tends to elicit stronger empathic and personal distress responses than more detached instructions.

Participant reactions also align with biological and moral psychology findings. Most commonly, participants recognised the significance of the maternal-filial bond for animal well-being and expressed concern over its disruption. Their perceptions echo biological studies showing that breaking this bond causes distress in animals (Meagher *et al.* 2019), as well as research on public attitudes that highlights widespread concern regarding such practices (Boogaard *et al.* 2010; Ventura *et al.* 2013; Hötzel *et al.* 2017). The disruption of the maternal-filial bond may be a powerful perspective-taking promoter, as indicated by participants relating how they would feel if separated from their kin. Moreover, the emergence of self-other overlap and distress during perspective-taking is supported by psychological constructs like self-other merging (Schultz 2001; Ahn *et al.* 2013) and is consistent with literature showing that personal distress can interfere with decision-making when emotional engagement is high (Ladak *et al.* 2023).

These empathetic and sympathetic reactions have the potential to influence decision-making. Self-other overlap, empathy and sympathy for others can predict prosocial intentions (Cialdini *et al.* 1997; Galinsky & Moskowitz 2000); findings suggest that fostering these emotional responses may help create psychological conditions to considering animals’ perspectives in decision-making.

For some participants, mostly in the video condition, cow-calf separation was felt to be particularly distressing. In the video condition, participants reported feeling “shocked”, “disturbed”, and psychologically uncomfortable, indicating their experience of sympathetic distress. These reactions may also interfere with decision-making, as the participants’ motivation to make an accurate decision will compete with their motivation to reduce their own distress. Distress avoidance might also explain the reactions of some participants who expressed challenges in taking the perspective of the cow and calf. By denying animals the possession of morally relevant qualities, such as the capacity for suffering, participants may diminish the animals’ moral standing, making it easier to justify their use and ill-treatment (Loughnan *et al.* 2010; Bilewicz *et al.* 2011; Bratanova *et al.* 2012). Distress avoidance may also explain the reactions of participants who were hesitant to make a final decision about the appropriateness of cow-calf separation, asking for additional information; these participants may have been avoiding resolving the dissonance between their (or their society’s) consumption of animal products and their own discomfort with the practice that they had witnessed.

While perspective-taking has been shown to improve attitudes toward non-human animals through empathy and self-other overlap, it does not always lead to stronger moral shifts. As Ladak *et al.* (2023) suggest, empathetic distress in perspective-taking can overwhelm individuals, preventing deeper engagement with the animal’s perspective. Alternatively, remaining objective can help manage emotional overwhelm but may reduce the connection needed to appreciate the animal’s experience. Both approaches require a balance, one that mediates distress in a way that enables meaningful engagement with the perspectives of others.

### External factors influencing perspective-taking

#### Video intervention

Both the audio and video participants expressed sadness related to cow-calf separation, either as their own personal feelings or via empathy for the animals, but these emotional accounts were more detailed in the video groups. According to some participants in the video groups, the video enhanced their sense of emotional connection to the animals’ experiences, making their perspectives easier to imagine. This ability to imagine the animals’ perspectives was reflected in participants’ responses in the video treatment, who engaged in broader speculation of the animals’ experiences. For example, Luma’s refusal to eat after her calf’s removal was seen by some as an expression of longing or grief. This act of refusal was perceived as Luma asserting her agency in an environment that limited her choices.

Heightened perspective of participants in the video treatment may have been due to increased vividness compared to the audio condition. Past research has investigated how varying levels of vividness contribute to perspective-taking and environmental attitudes. Increasing vividness of visual cues related to environmental practices, either with images (Sevillano *et al.* 2007), films (Shelton & Rogers 1981), or through the use of virtual reality (Ahn *et al.* 2016), may promote concern for the biosphere. Combined, these findings suggest that vivid portrayals of practices can shape perspective-taking.

#### Anthropocentric norms

Participants expressed a handful of reasons why they found it challenging to take the animals’ perspectives, including their lack of encouragement or opportunity to understand animal perspectives and the lack of information to guide perspective. For many participants, the challenge rested in feeling uncertain about inferring states in cows. This concern for anthropomorphism is consistent with the taboo against projecting human characteristics onto non-human entities that is widespread in the biological and psychological sciences (Humphreys 2023). Participants in this study were recruited through the Psychology Department Research Pool and thus had all taken at least one course in psychology. While the scientific and philosophical debate around the appropriateness of anthropomorphising non-human animals remains thorny (see de Waal 1999; Burghardt 2007; Webb *et al.* 2023), focusing on animals’ perspectives provides an opportunity to transcend anthropocentric normative assumptions that are prevalent in academic settings (Rosenfeld 2021; Thomsen *et al.* 2023). Luma and her calf’s story, captured in the film, was an effort by the filmmaker to allow the animals to share their own narrative. Seeing the experience of Luma and her calf, in context, elicited a greater depth and range of perspectives on the experience of cows and calves, but also elicited concerns regarding anthropomorphism in some participants.

### Limitations and future directions

Efforts to meaningfully incorporate animals’ perspectives into decision-making face important limitations and point toward future directions. Progress depends on at least three interrelated components: decision-makers’ motivation to engage with animals’ perspectives; access to reliable and contextually rich information about animals’ experiences; and careful calibration of psychological distance such that empathy is facilitated without provoking distress-driven avoidance. In what follows, we consider each of these components in turn, grounding them in the present findings, while outlining associated limitations and directions for future research.

First, decision-makers must be motivated to engage with the animal’s perspective. This study focused on understanding the variation in this motivation, addressing participants’ willingness to take animals’ perspectives, rather than the accuracy of their speculations about animal experiences. The conclusions of this study are limited by the participant sample, the sample stimuli, and specific topic considered. A convenience sample of undergraduate psychology students was recruited, most of whom were young and female. A limitation is that we did not systematically assess participants’ prior experience with farming; in some instances, participants spontaneously discussed their familiarity with farming practices and these discussions suggested limited familiarity with conventional dairy farming practices. The results would have likely been different if the sample included farmers or others more knowledgeable about cow-calf separation. Indeed, other samples could yield interesting insights into how different people take the perspectives of animals, including students outside of psychology and non-university educated participants. In addition, given that participants in this study often considered the practice of cow-calf separation in relation to the experiences of human mothers, future work should consider the views of both mothers and non-mothers.

The results of our study suggest that showing a video of cow-calf separation helped perspective-taking by some participants. In this study we compared only two depictions of animals’ experiences: a text-based description and a short film clip intentionally produced to portray an individual animal with empathy and dignity. The video stimulus in this study was selected, in part, because it provided a presentation of the cow-calf separation without narration. These choices represent two points in a much larger design space, and our findings should not be interpreted as evidence that video (or any specific medium) is inherently superior. Other video stimuli would likely influence viewers’ emotional experiences and ability to perspective take. Future work should systematically vary candidate elements that could underlie ‘evocativeness’, such as sensory richness, perceived proximity, narrative structure, perceived individuality, perceived agency, and opportunities for behavioural contingency (e.g. animal responses vary in relation to human actions) to identify which features most reliably facilitate perspective-taking and whether the effects persist. Such studies would help clarify how animals’ perspectives become salient in decision-making contexts.

Second, in addition to people being motivated to take the perspectives of non-human animals, effective decision-making will require reliable information (knowledge and evidence) about the animals’ experiences. Much research in animal welfare science attempts to provide this understanding of what animals want and how important it is to them to achieve this. Although we did not directly test this knowledge dimension, our findings suggest that incorporating animals’ perspectives will require providing participants with detailed, context-specific information about the conditions animals experience. For example, to better engage decision-makers, interventions should highlight animals in environments that allow for agency and expression. Some study participants discussed animal experiences that are not well studied in the welfare literature, such as trust and grief, suggesting the need for further work by animal welfare scientists. An important future direction concerns the influence of perspective-taking on decision-making (e.g. weighting of animal interests, effect on policy).

An original goal of our study was to examine the decision of participants to support or not support the practice of cow-calf separation. However, most participants expressed hesitation or frustration in making this decision due to feeling like they did not have sufficient information, including wanting more information on the relevant science, how accurate their interpretations were, and a desire to have other stakeholders in the conversation representing their views. Whether or not this desire for information was prompted by the perspective-taking exercise is unclear. It may be the case that perspective-taking caused people to increase the standard of evidence required when considering a practice, perhaps because participants better understood the consequences of their decision. Beyond understanding what prompts requests for more information, it will also be important to understand what kinds of information would satisfy participants. Indeed, future studies may wish to ask participants: “What information do you need to better understand the perspective of animals?” or “If you were asked to speak for these animals in decision-making, what would you say?” Future research could also explore how perspective-taking is prioritised in decision-making and influenced by stakeholder compositions.

Third, to fully address how decision-makers engage with animals’ perspectives it is necessary to consider the balance between psychological distance and distress, as closing this distance facilitates empathy (Ahn *et al.* 2013) but being *too close for comfort* can lead to avoidance reactions. Understanding the intricacies of this balance marks an important direction for future research. Focusing on cows may have made perspective-taking easier for some participants. Indeed, many participants struggled with the distress of cow-calf separation which, while not directly measured, likely evoked empathetic distress — a self-referential reaction to others’ suffering (Singer & Klimecki 2014). Our results anticipate that efforts to incorporate animals’ perspectives must include the presentation of rich, contextually accurate depictions of the realities that animals face. Future studies could explore how different interventions, such as structured narrative framing, or utilising storytelling and technology, can reduce defensive responses and encourage more helpful actions (Singer & Klimecki 2014; Ahn *et al.* 2016; Nussbaum 2022).

This study focused on cows, mammals that form strong maternal-filial bonds that seem to resonate with humans. A focus on cows may have facilitated perspective-taking, at least for some participants. For species from which humans feel more distant from, perspective-taking responses may weaken, and when this gap is large perspective-taking instructions may prove ineffective (Ladak *et al.* 2023).

A final limitation arises from our choice of methods. The focus groups began with participants responding to a survey that gathered responses specific to their perspective, but during the group discussion participants shared their views, and people were likely influenced by their group-mates; evidence of a social desirability bias (Bergen & Labonté 2020) could be seen in some responses (e.g. in FG10 video, where one respondent’s views seemed to sway others in the group). Focus group facilitators also had views on the practice of cow-calf separation and the capabilities of cattle; although they took an impartial stance during the discussions and provided information based on their academic understanding of the issues, it is possible that their views still influenced participants (Acocella 2012).

### Animal welfare implications

The results of our study show that participants vary in perspective-taking and suggest that contextual factors (such as seeing a video shot from the cow’s perspective) can facilitate perspective-taking by some participants. Anthropocentric decisions shape the lives of animals used on farms, in laboratories, and for other reasons. Animals’ welfare stands to improve when animal interests are identified and are given weight in decision-making.
